# Subnanosecond Electrical Control of Dipolariton-Based Optical Circuits with a Few Femtojoule per Bit Power Consumption

**DOI:** 10.1021/acs.nanolett.5c02461

**Published:** 2025-08-07

**Authors:** Dror Liran, Kirk Baldwin, Loren Pfeiffer, Hui Deng, Ronen Rapaport

**Affiliations:** † Racah Institute of Physics, 450737 The Hebrew University of Jerusalem, Jerusalem 9190401, Israel; ‡ Department of Electrical Engineering, 6740 Princeton University, Princeton, New Jersey 08544, United States; ¶ 1259 University of Michigan, Ann Arbor, Michigan 48109, United States

**Keywords:** Exciton-Polaritons, Electrical-modulation, Waveguide, Quantum-optics

## Abstract

The next generation of photonic circuits will require programmable, subnanosecond, and energy-efficient components on a scalable platform for quantum and neuromorphic computing. Here, we present subnanosecond electrical control of highly nonlinear light–matter hybrid quasi-particles, called waveguide exciton-dipolaritons, in a highly scalable waveguide-on-chip geometry, and with extremely low power consumption. Our device performs as an optical transistor with a GHz-rate electrical modulation at a record-low total energy consumption <8 fJ/bit and a compact active area of down to 25 μm^2^. This work establishes waveguide-dipolariton platforms for scalable, electrically reconfigurable, ultralow power photonic circuits for both classical and quantum computing and communication.

## Graphical abstract

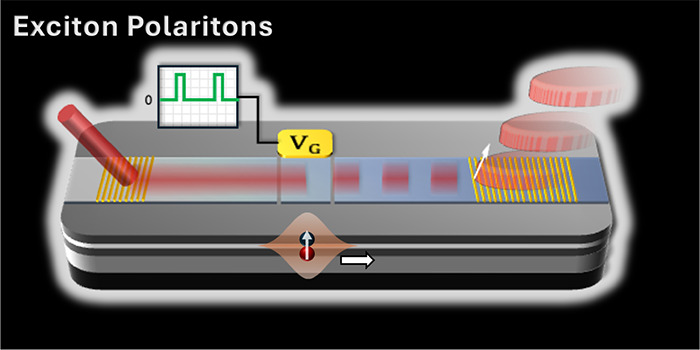

Integrated photonic circuits are the most promising platform for the rapidly developing fields of optical information processing,^1–5^ including classical and quantum simulators and computers,^6,7^ as well as neuromorphic calculators.^8,9^ A crucial requirement of an integrated photonic platform^8,10^ is the ability of subnanosecond *electrical* control of individual nodes with ultralow power consumption per node per operation.

Exciton polaritons (EP),^11^ resulting from the strong coupling of confined photons and two-dimensional excitons, are excellent building blocks for photonic circuits. They can be directly addressed due to their photonic part, and they have stronger optical nonlinearities due to their excitonic part. Recent demonstrations of subnanosecond all-optical control of EP in microcavities, including optical switching,^12–19^ EP condensate-based photonic lattice simulators,^6,7,20^ and EP based neuromprphic computing^9,21^ are just a few examples of the promise of EP platforms.

Yet, for modern complex photonic circuitry, highly desirable are electrically controlled photonic nodes that are densely integrated into monolithic chip geometries. For microcavity-based EP elements, only optical control has been demonstrated so far; significant optical power is required per node, typically exceeding 10^3^J/node/operation at 1 GHz;^12^ scaling up layers of nodes for deep circuits is also a major challenge, as microcavities cannot be easily stacked.

Alternatively, monolithic chip-integrated waveguide exciton-polaritons (WEP)^22,23^ are well suited for scalable EP circuits. Several key elements have been demonstrated recently.^24–29^ An important step toward locally reconfigurable and scalable waveguide exciton-polariton (WEP) circuits is the realization of *electrically gated dipolar WEP* (DWEP) structures, where a top gate applies a perpendicular electric field to wide quantum wells (QWs) inside the waveguide, inducing a quantum-confined Stark effect and voltage-controlled exciton dipoles.^23^ These dipoles exhibit strong dipole–dipole interactions and screening effects, enhanced further by the ultralight effective mass of the polaritons,^30^ resulting in record-high effective nonlinearities—up to 2 orders of magnitude larger than in unpolarized polaritons^26,27,31,32^ —and enabling key demonstrations such as electrically controlled few-photon transistors.^28^ Remarkably, electrically tunable quantum correlations and a partial 2-photon blockade were recently demonstrated, showing potential for realizing DWEP for electrically switchable, universal 2-photon gates.^33^

Here, we demonstrate a fast, subnanosecond temporal control of such nonlinear DWEP-switches operating at record-low powers of a few Femto-Joule/bit and a very small footprint of <50*μm* ^2^ per node, a significant step toward complex optical circuitry based on WEP, toward universal photonic computation.

The device used in our experiments (Figure 1(a)) is a 200 μm long and 5 μm wide waveguide channel, optically defined by an indium tin oxide (ITO) strip, with two Au diffraction gratings for input and output at either end. The waveguide itself is constructed out of an *Al*_0.4_ *Ga*_0.6_ *As* core with 12 embedded 20 nm-wide *GaAs* quantum wells (QWs). For details on the full sample fabrication, see ref.^28^ The strong interaction of the transverse-electric (TE) polarized waveguide mode with the heavy-hole (*hh*) and light-hole (*lh*) excitons leads to the formation of three polariton modes: Lower-Polariton (LP), Middle-Polariton (MP), and Upper-polariton (UP).^23^ Each polariton mode is a superposition of the bare TE-photon and the two excitons, represented as |ψ_*pol*_(β)⟩^*i*^ = χ_*ph*_^*i*^(β)|ψ_*ph*_⟩ + χ_*hh*_^*i*^(β)|ψ_*hh*_⟩ + χ_*lh*_^*i*^(β)|ψ_*lh*_⟩, where *i* = LP, MP, and UP respectively, and the Hopfield coefficients satisfy |χ_*hh*_|^2^ + |χ_*lh*_|^2^ + |χ_*ph*_|^2^ = 1. The total exciton fraction of the polariton is defined here as |χ_*X*_(β)|^2^ = 1 – |χ_*ph*_(β)|^2^. LP-polaritons having |ψ_*pol*_(β)⟩^*LP*^, *E*_*LP*_(β) can be excited at the left grating by a resonant laser (Ti:Sa, CW) with its energy and incidence angle matching the desired position on the polariton dispersion. We define a relative DWEP dispersion *E*(β) = *E*_*LP*_(β) – *E*_*hh*_, measured with respect to the unbiased *hh*-exciton energy, *E*_*hh*_. Such dispersions are shown in Figure 1(d, e). The output signal is collected from the right grating and imaged onto a spectrometer. Cross-polarization is employed in both the excitation and emission paths to isolate the polariton emission from the scattered laser light. Additionally, spatial filtering further reduces laser scattering (see Figure S2 in the SI - Supporting Information). To allow independent control of the electric field in each section, the ITO strip that defines the optical waveguide^28^ is divided into three sections by *a* < 1*μm* gap in the electrode. The middle section, named the ”gate”, is electrically biased. The gate is 10 μm long and is centered between the input and output gratings. The outer sections (hereafter the ”channel”) are held at zero bias.

**Figure 1:**
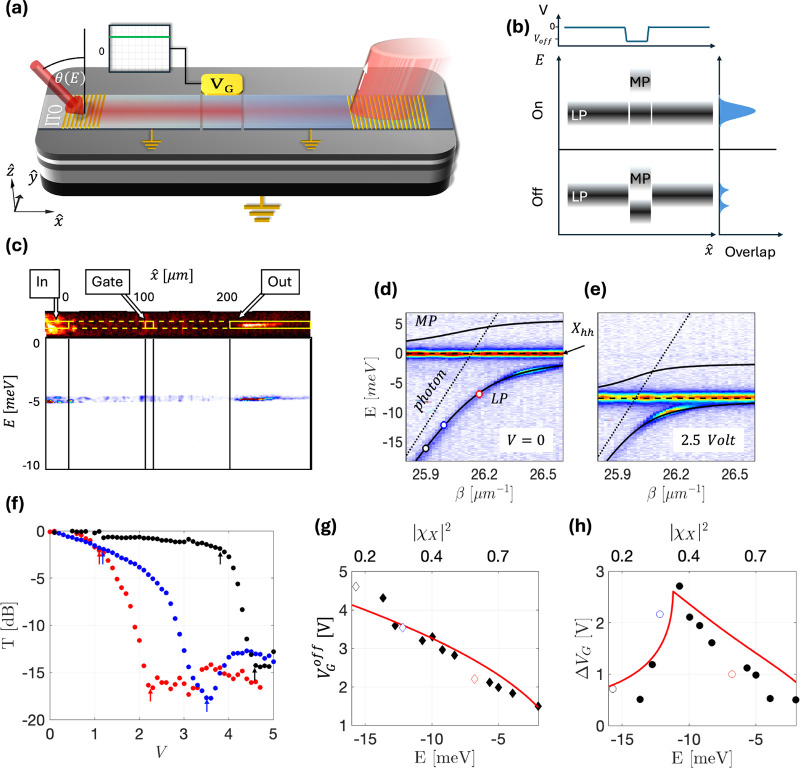
Setup and electric field dependence of experiments with gated-DWEP devices: (a) Schematic diagram of the device structure, with the ITO electrode divided into three parts: the gate *V*_*G*_ and the unbiased channel. The incident laser excites the sample at θ­(*E*) to satisfy the WEP dispersion. The transmitted light is a function of the field amplitude *V*_*G*_. (b) Energy diagram for the polariton switching. Top Panel: schematic of the voltage values along the waveguide. Middle panel: “on” - the gate is flat with the channel allowing the polaritons to transmit. Bottom panel: “off” - the gate is biased such that the states from the channel meet the LP-MP gap in the gate, and the polaritons do not transmit. (c) Top panel: a real-space image of the WEP experiment. The laser is injected in the left grating, and the WEP signal couples out from the right grating. Bottom panel: a spectrally resolved imaging measurement of the WEP. (d and e) Measured WEP dispersion at *V*_*G*_ = 0 and *V*_*G*_ = 2.5 V/μm, respectively. The plots show the energy *E* as a function of the wavevector (β). The lower polariton (LP) and middle polariton (MP) model fits are indicated by solid black lines. A flat dashed line marks the heavy-hole exciton, and a dotted diagonal line marks the bare WG-photon. The colored dots in panel d correspond to the three curves in panel f. (f) The transmission in the channel as a function of *V*_*G*_ for three different WEP excitation energies *E* = −7, −12, −16 meV marked in panel d respectively. The arrows mark the points defined as the on–off transition. (g) The voltage value (*V*_*G*_) of the first minimum in the transmission. The values for the curves in (f) appear in the corresponding color. The red line plots the voltage value required for the exciton to shift such energy. (h) The voltage difference (Δ*V*_*G*_) between the first minimum in transmission and a transmission of 0.6. The two colored points represent the corresponding transitions marked in panel f by colored arrows. The red lines mark the model prediction.

When a field is applied to the gate, the DWEP transmission between the input and output gratings decreases due to the reduced overlap of the DWEP density of states under the gate and in the channel;^28^ an illustration of the mechanism is presented in Figure 1(b), where minimum transmission occurs when the gate voltage *V*_*G*_ is shifting the LP energy by exactly one Rabi frequency Ω­(*V*_*G*_), i.e., Δ*E*(Δ*V*_*G*_) = Ω­(*V*_*G*_), where (Δ*V*_*G*_) is the voltage difference for switching between on and off states. Figure 1(f) plots the DWEP transmission *T* (normalized to its maximum) as a function of the gate voltage (*V*_*G*_) for three different excitation energies *E* = −7, −12, −16 meV. Arrows on each curve indicate the *T* = 0.6 (−2.2 dB) and minimal transmission points.

Such transmission curves are then used to extract *V*_*G*_(*E*) required for blocking the DWEP propagation for different polariton excitation energies, as is plotted in Figure 1(g). From the same transmission curves we also extract Δ*V*_*G*_(*E*), Figure 1(h). Interestingly, Δ*V*_*G*_(*E*) shows a nonmonotonic behavior. This behavior arises from the nonlinear dispersion of the DWEP, *E*(β). For simplicity, we employ a two-mode WEP model (see details in the SI), yielding the following expression:

$$\Delta V_{G}=\{\begin{array}{ll} V_{G}^{\mathrm{off}}\left(1-\sqrt{\frac{E{\left(\beta \right)}_{0}+\Omega \left(V_{G}\right)}{E{\left(\beta \right)}_{0}}}\right), & E{\left(\beta \right)}_{0}>\Omega \left(V_{G}^{\mathrm{on}}\right)\\ \approx V_{G}^{\mathrm{off}}, & |E{\left(\beta \right)}_{0}|<\Omega \left(V_{G}^{\mathrm{on}}\right) \end{array}$$ 1

where *E*(β)_0_ ≡ *E*(β, *V*_*G*_ = 0) is the energy of the resonantly injected polariton, $V_{G}^{\mathrm{off}}=\sqrt{\frac{-E{\left(\beta \right)}_{0}}{\alpha^{\prime }}}$ is the voltage required to shift the exciton line to the polariton injection energy, and *α′* = 1.53*meV*/*V* ^2^,^28^ is the experimentally extracted electric polarizability of the QW hh-exciton. The model agrees well with the data, as is shown by the red lines in Figure 1(g,h).

To test the frequency response of the system, we injected WEP resonantly through the input grating using a CW laser, while modulating the gate voltage using a square electrical pulse *f*(*t*) with a nanosecond rise and fall times. The experiment was repeated with different periods and duty cycles, as depicted schematically in Figure 2(a). The field amplitudes and offsets of the electrical modulation were selected based on the DC measurements presented in Figure 1 (f-h), and can be represented by *V* (*t*) = *V*_*G*_^*off*^ + Δ*V*_*G*_ × *f*(*t*). The output signal was imaged onto a streak camera. Such Streak images for two different modulation frequencies are presented in Figure 2(b,c). Figure 2(d,e) presents the time-domain DWEP transmission of single pulses (blue dots) plotted on top of the modulating electric field (solid green line) for WEPs excited at – 13.7, – 4 *meV*, corresponding to excitonic fractions of |χ_*X*_|^2^ = 0.26, 0.77 respectively. The black lines indicate the ”on” (solid) and ”off” (dashed) states. The transmission data is fitted with a pulse function (red line):

$$T\left(t\right)=T_{\mathrm{on}}/2\times \left(\tanh\left(\frac{t-t_{r}}{\tau_{r}}\right)-\tanh\left(\frac{t-t_{f}}{\tau_{f}}\right)\right)+T_{\mathrm{off}}$$ 2

where τ_*r*_ and τ_*f*_ are the rise and fall times, respectively.

**Figure 2:**
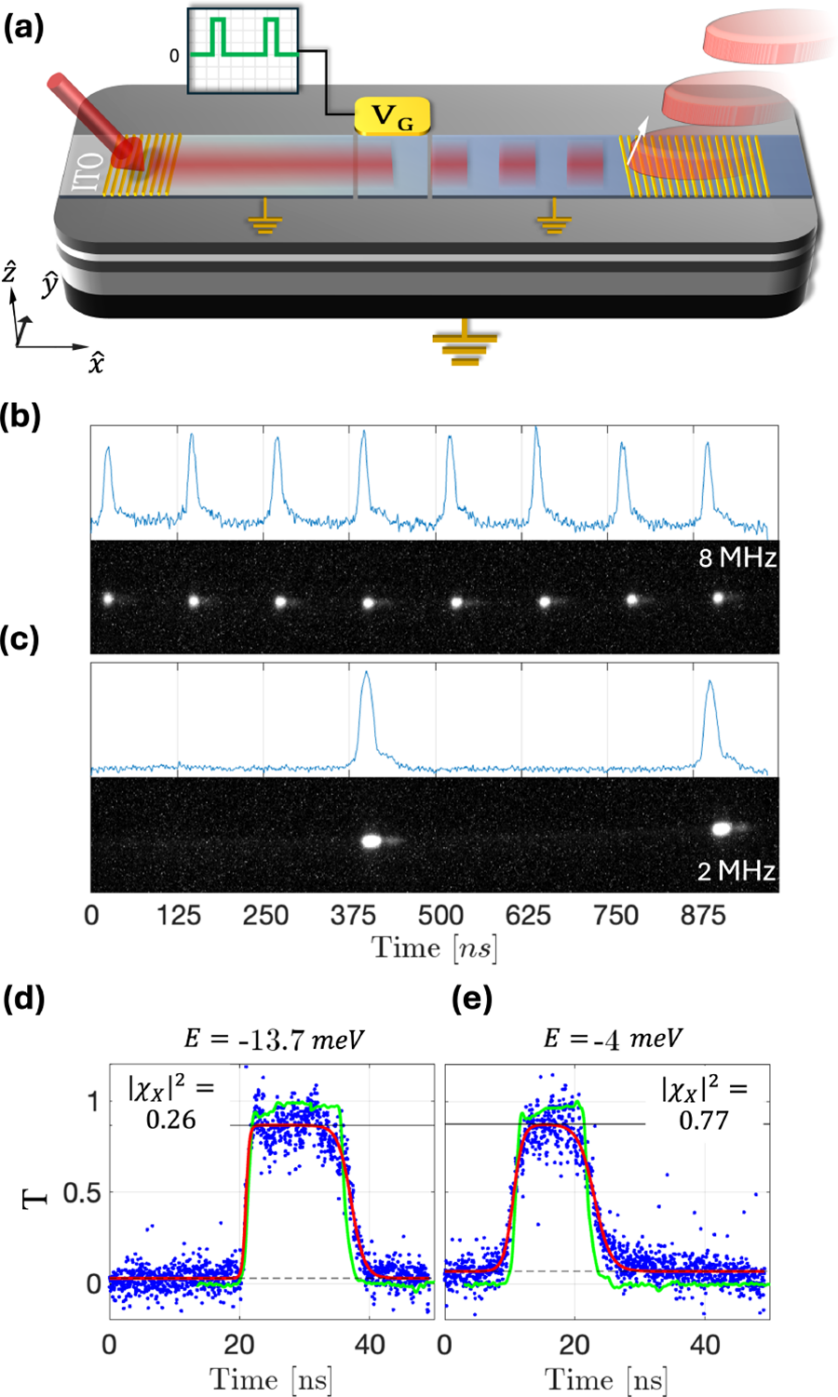
Pulse Modulation: (a) Illustration of the device response to a square voltage modulation of the gate. (b and c) Time-resolved measurement of the transmitted signal at 8 and 2 MHz, respectively. The bottom panel plots the raw data from the streak camera, while the top panel plots the integrated amplitude. (d and e) Normalized time-domain pulse transmission (blue dots) for two different polariton energies *E* = −13.7 meV and *E* = −4 meV, plotted along the normalized electric signal input (green line). The data is fitted to a pulse function (red line). The black lines mark the “on” (solid) and “off” (dashed) states.

From such fitting as above, we extracted τ_*r*_, τ_*f*_, as well as the extinction ratio (ER), *ER* = *T*_*on*_/*T*_*off*_, for various excitation energies, for electrical pulses with a 4 MHz carrier frequency, and pulse durations of 15–20 ns. The extracted rise and fall times are plotted in Figure 3(a,b), and are both smaller for lower energy DWEPs, corresponding to more photon-like polaritons. Rise times as short as 0.5 ns and fall times as short as 1 ns are measured. As seen from Figure 2(d,e), the optical signal of photon-like DWEPs follows the electrical pulse. This means that the electrical pulse generator still limits the rise and fall times and not the WEP system intrinsically. Therefore, we conclude that our devices can operate at > GHz bandwidth.

**Figure 3:**
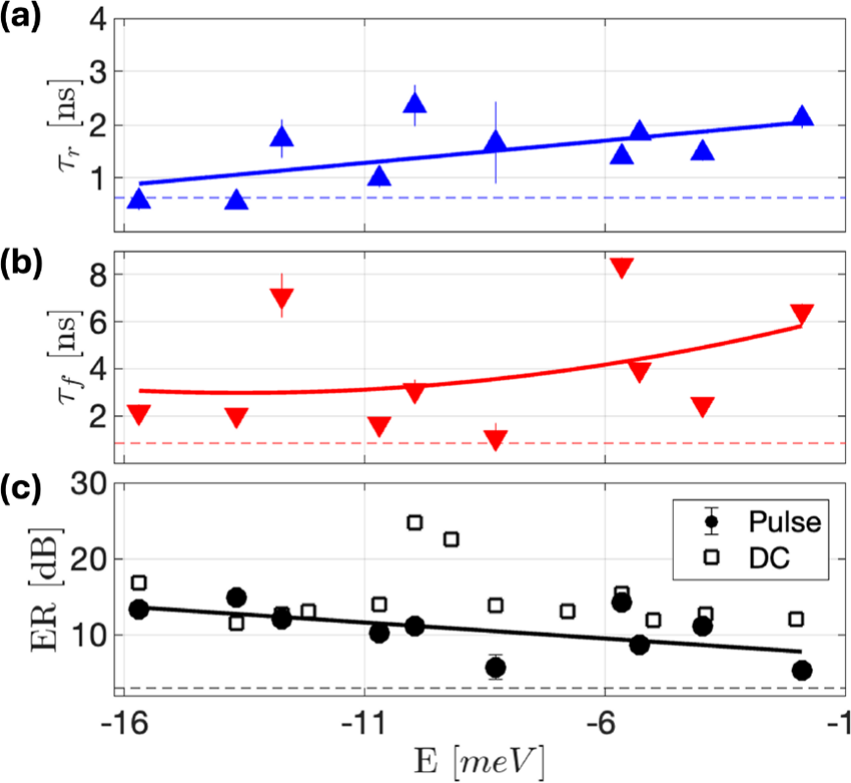
Bandwidth, extinction, and power consumption: (a and b) Extracted transition times (rise and fall) as a function of the excited WEP energy. The lines are guides to the eye. The error bars represent the fit uncertainty values. The dashed line marks the input voltage transition time. (c) Extinction ratios as a function of excited WEP energy. The line is a guide to the eye. A dashed black line marks the minimal required ER (3 dB). The corresponding DC extinction ratio is also plotted with empty squares.

The extinction ratio is shown for various DWEP energies in Figure 3(c,d). Again, more photon-like DWEPs tend to have a higher extinction ratio, indicating more efficient switching. Importantly, these photon-dominated states also have faster propagation in the WG, lower propagation loss, and better in- and outcoupling efficiencies. *ER* values as high as 15 dB are measured, limited only by the SNR of the Streak camera. To demonstrate that the actual ER values are higher, we plot the *ER* for the DC case, which had a better SNR of up to 25 dB.

The energy consumption for our device is composed of two factors, the energy of photons in a cycle and the electrical energy to operate the switching, given by the current–voltage product in a cycle. To estimate this, we measured the current of the device during operation, with *V*_*G*_^*off*^ = 3.1*V* and Δ*V*_*G*_ of 2.25, we measured currents of *I* = 0.46 and 0.85 μA, respectively. For a duty cycle of R = 50*%* and frequency of *f* = 1 GHz, the electrical energy consumption per operation is less than 6.1 fJ/bit. Details appear in the SI. The optical power required for the nonlinear DWEP-transistor operation is 1.2 fJ per cycle at 1 GHz (see SI). Finally, electrical and optical energy consumption adds up to less than 8 fJ per cycle at 1 GHz for a fully functional electrically modulated optical transistor device.

We demonstrated DWEP signal modulation with a bandwidth exceeding 1 GHz, primarily constrained by the limitations of the electrical pulse generator and the electronic circuitry, together with a very high extinction ratio. A proper electrical design can significantly improve the bandwidth, as was demonstrated in GaAs-based electro-optical devices that have achieved modulation frequencies up to 100 *GHz*.^38^ To achieve a higher bandwidth, future designs will include impedance matched^39^ Ohmic contacts to the GaAs waveguide as shown in ref.^38^ This is expected to push the currently limiting electrical bandwidth to as high as 100 GHz, which should allow a modulation rate limited only by the intrinsic polariton constrains With a minimal footprint (fabricated down to 25 μm^2^), such elements are well suited for high-density photonic circuitry. The footprint could be further reduced by shortening the gate length to a few times the WEP wavelength in the medium (λ ≃ 240 nm), and decreasing the channel width to ∼0.5 μm by side etching,^24,26^ resulting in an overall footprint as small as ∼1 μm^2^ per node. Such bandwidth and minimal footprint are also available with plasmonics-modulators, however with a much higher insertion loss, see Table 1.

**Table 1:** Integrated Electro-optic Modulators

Mechanism	Material	Power consumption [fJ/bit] @ 1 GHz^a^	ER [dB]	Footprint [μm^2^]	Insertion Loss [dB]	Bandwidth GHz
DWEP (this work)	GaAs	<8^b^	50(25)^c^	25^d^	∼2	2
Pockels effect (MZI^e^)^34^	LN	26	30	10^4^	<1	100
Carrier injection^35^ ^g^	Si	500	8	10^3^	>1	40
Plasmonic (MZI)^36,37^	Si and Au	2750	25^f^	∼10	5	110
MC EP (all optical)^12^	GaAs^h^	1980	12	1.6 × 10^3^	10	100

a The power consumption was rescaled to a bit rate of 1 GHz with duty-cycle of 0.5.

b See the SI for a full derivation.

c See DC measurements of ER in Figure 3, limited by the SNR.

d The results presented here are on a 50 μm^2^ device. Similar results were also measured with a 25 μm^2^ device (not shown).

e Mach-Zender interformeter.

f The device with the lowest power consumption.

g Loss of 5 dB per element.

h Can also be realized in many other material systems, see ref 14.

Remarkably, our device demonstrates an overall power consumption of about 8 fJ/bit, which, as far as we know, sets a record compared to other platforms, as detailed in the Table 1. We emphasize that low power consumption per node per operation is a crucial factor in large-scale fast circuitry, including those designed for classical neuromorphic computing as well as for quantum computing. Both cases require a large amount of reconfigurable elements.^40^ Further reduction in power consumption can be achieved by minimizing tunneling between the top and bottom contacts and reducing the surface area of the electrical contacts, which in our current design constitute the most significant contributors to leakage currents.

Finally, the same device displays an electrically tunable, optical nonlinearity which is, as far as we know, the highest of any exciton-polariton-based system^33^ The very high electrically controlled polariton nonlinearities enable operation at very low powers, thus allowing us to avoid self-phase modulation and other nonresonant nonlinearities which exist in such systems.^26,41^ As was mentioned before, this led to a demonstration of a fully operational optical transistor,^28^ and to quantum correlations at the two-photon level,^33^ which can be controlled very accurately by the applied electric field. The current demonstration of >GHz modulation of an optical node, will allow a reconfigurable polariton-based optical circuitry, where the linear and nonlinear function at each node can be controlled separately and be reconfigured for each operation, making electrically polarized DWEP an excellent candidate for reconfigurable deep optical circuits for either neuromorphic or quantum processors in a monolithic platform.

## Supplementary Material



## Notes

Supporting calculations, a description of the optical setup, and raw data images for DC and pulsed experiments (PDF)
