# Supplementary material for: Subnanosecond Electrical Control of Dipolariton-Based Optical Circuits with a Few Femtojoule per Bit Power Consumption
Source: Nano Lett. 2025 Aug 7;25(33):12503–8. doi: 10.1021/acs.nanolett.5c02461 (PMC12371871; doi:10.1021/acs.nanolett.5c02461)
Supplement: Supplementary file 1 [file nl5c02461_si_001.pdf]

Supporting Information for: Sub-nanosecond electrical control of dipolariton-based optical circuits with a few femto-joule per bit power consumption

Dror Liran,† Kirk Baldwin,‡ Loren Pfeiffer,‡ Hui Deng,¶ and Ronen Rapaport*,†

†*Racah Institute of Physics, The Hebrew University of Jerusalem, Jerusalem 9190401, Israel*

‡*Department of Electrical Engineering, Princeton University, Princeton, NJ, 08544 USA*

¶*University of Michigan, Ann Arbor, MI 48109, USA*

E-mail: ronenr@phys.huji.ac.il

The Supporting Information contains supporting calculations, a description of the optical setup, and raw data Images for DC and pulsed experiments.

Voltage modulation for DWEP Switching - model

Here we develop a simple model for the voltage modulation required for switching between full transmission T_{on} minimal transmission T_{off}. Minimal transmission occurs at a gate voltage amplitude V_G that shifts the polariton eigenenergies under the gate such that the incoming WEP with a given β, $E_{LP0}(\beta)$ will be at the middle of the LP-MP gap under the gate.[1] This corresponds to:

$$E_{LP0} \simeq E_{hh} - \alpha'(V_G^{off})^2, \tag{S1}$$

and

$$E_{LP0}+\Omega(V_G^{on})\simeq E_{hh}-\alpha'(V_G^{on})^2, \tag{S2}$$

where $\alpha' = 1.3/0.85 = 1.53meV/V^2$ is the voltage-dependent polarizability as calculated in Ref.[1]

The transmissive state requires a voltage amplitude modulation that induces an energy shift smaller by $\Omega(V_G^{on})$, compared to the blocking voltage, $V_G^{\text{on}} < V_G^{\text{off}}$.

Inverting the equations, the minimal transmission for polariton injected in energy $E_{LP0}(\beta)$ is given at

$$V_G^{off}=\sqrt{\frac{E_{hh}-E_{LP0}(\beta)}{\alpha'}}. \tag{S3}$$

Thus, the maximal shift needed for blocking the signal is is given by V_G^{off}. However, since the transmission only drops when the LP-MP gap is shifted to the vicinity of the desired energy, i.e. at an energetic distance of Ω, this gives a voltage difference of:

$$\Delta V_G=\begin{cases} V_G^{off}\left(1-\sqrt{\frac{E_{hh0}-(E_{LP0}(\beta)+\Omega(V_G^{on}))}{E_{hh0}-E_{LP0}(\beta)}}\right), & E_{hh0}-E_{LP0}>\Omega(V_G^{on}) \\ \approx V_G^{off}, & E_{hh0}-E_{LP0}<\Omega(V_G^{on}) \end{cases} \tag{S4}$$

Where $\Delta V_G = V_G^{off} - V_G^{on}$, and V_G^{on} is given by the voltage where we expect to have transmission

$$V_G^{\text{on}}=\begin{cases} \sqrt{\frac{E_{hh0}-(E_{LP0}(\beta)+\Omega(V_G^{on}))}{\alpha'}}, & E_{hh0}-E_{LP0}>\Omega(V_G^{on}) \\ \to 0, & E_{hh0}-E_{LP0}<\Omega(V_G^{on}) \end{cases}. \tag{S5}$$

We note that the Rabi frequency $\Omega(V_G)$ decreases with increasing voltage,[2] and can be described by $\Omega(V_G) = \Omega(V_G = 0) \times \exp(-V_G^2/2\sigma_\Omega^2)$, where σ_Ω and $\Omega(V_G = 0)$ are fitting parameter for our system, with values of 11 meV and 4 V, respectively. The experimental data and the model are shown together in Fig. 1(h).

Energy per Operation Calculation

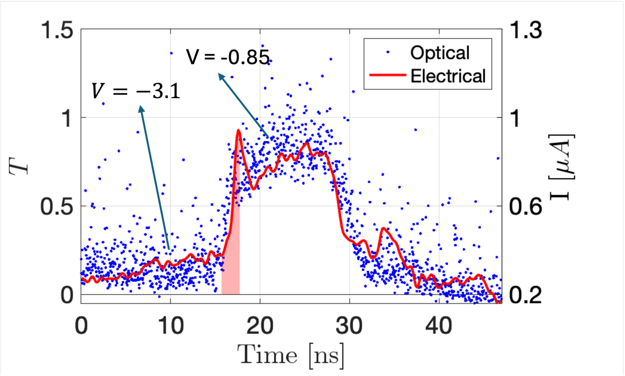

Figure S1: Time-resolved measurement of the electrical current at $E = -10.5meV$ (*red line*) and optical transmission (*blue dots*) as a function of time. The off voltage is $V = -3.1\,\mathrm{V}$ with a ramp of $\Delta V = 2.25\,\mathrm{V}$ for transmission. This data was used to calculate the average energy per operation. The optical signal tracks the changes in the system, demonstrating the correlation between electrical and optical properties.

To estimate the energy per operation, we consider two scenarios. (1) The device is in an *on*-state all the time. This leads to a constant current (*DC*) of $I_{on} = 0.46\,\mu\mathrm{A}$ at $V_{on} = -3.1\,\mathrm{V}$. (2) The device is modulated between the *off* and *on* states with a 50%-50% duty cycle $f = 1\,\mathrm{GHz}$. Here the current alternates between I_{on}, V_{on} and $I_{off} = 0.85\,\mu\mathrm{A}$ at $V_{off} = -0.85\,\mathrm{V}$. For the *DC case*, the energy per operation is $U_{\mathrm{DC}} = 1.43\,\mathrm{fJ/opreation}$, at the 50%-50% duty cycle, the average current is $I_{\mathrm{avg}} = \frac{I_{on}+I_{off}}{2}$, and the average voltage is $V_{\mathrm{avg}} = \frac{V_{on}+V_{off}}{2}$. The energy per operation is then given by $E_{\mathrm{op,avg}} = 1.3\,\mathrm{fJ/opreation}$. The data on which these calculations are based are plotted in Fig.- S1. In addition, the energy cost of charging the gate capacitor can be estimated experimentally by $E_C = \int I(t)V(t)dt$, thus integrating over the shaded red area in Fig. S1 which yields $E_C < 4.8fJ$, with $V(t) = const = 3V$ as an upper bound. The nonlinear response of the system, as reported in Ref.,[1] requires $\sim$ 5000 photons for a 500 ps pulse, a 0.5 duty cycle at a 1 GHz repetition rate, to achieve the same DWEP density required for the transistor operation. The energy per pulse, E_{pulse}, can be calculated as the product of the photon energy (E_{ph}) and the number

of photons per pulse (N_{ph}):

$$E_{\text{pulse}} = E_{\text{ph}} N_{\text{ph}}. \tag{S6}$$

For photons with an energy of approximately 1.527 eV, this corresponds to a total of 1.2 fJ per cycle, highlighting the system's remarkable energy efficiency.

Finally, this yields an upper bound of $1.2 + 4.8 + 1.3 = 7.3 fJ$ per cycle.

Optical Setup

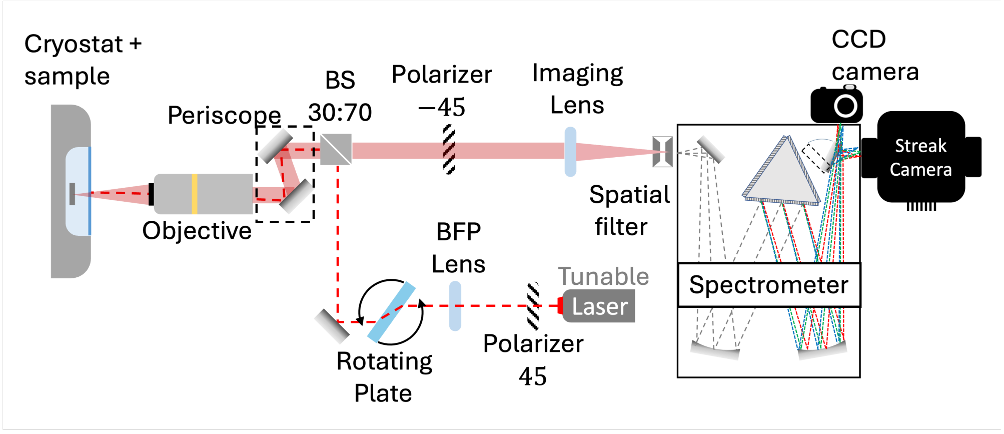

Figure S2: **The optical Setup:** A red dashed line marks the incident light, and a shaded red line marks the emitted light.

The optical setup, presented in Fig. S2, contains a CW Titanium-Sapphire Laser (model 3900 by spectra-Physics). The laser is focused on the back-focal plane (BFP) of the objective lens, at a spatial location such that it will enter the in-coupling grating at a desired single angle of incidence corresponding to the LP dispersion at a given energy. To choose the angle, we use a glass plate (about 5 mm thick), which deflects the beam to different positions at the BFP, i.e., different angles of incidence. Furthermore, the laser light is polarized at a 45° with respect to the in-coupling grating. The emission path includes cross-polarized light emitted at a −45° with respect to the out-coupling grating. This setup is designed to separate the emitted light from the scattered laser light. After the light is emitted from the sample, it is imaged in the entrance slit of a spectrometer. A spatial filter is used to filter out all other

emission except from the output grating coupler. The spectrometer has two outputs: a cooled CCD camera and a Streak Camera for imaging or time-resolved experiments respectively.

Data sets

Below, we plot the raw data used in the main text and data that supports the measurements in the main text. Figures S3 and S4 shows the Raw-data used for the analysis presented in Figures 1 and 2 .

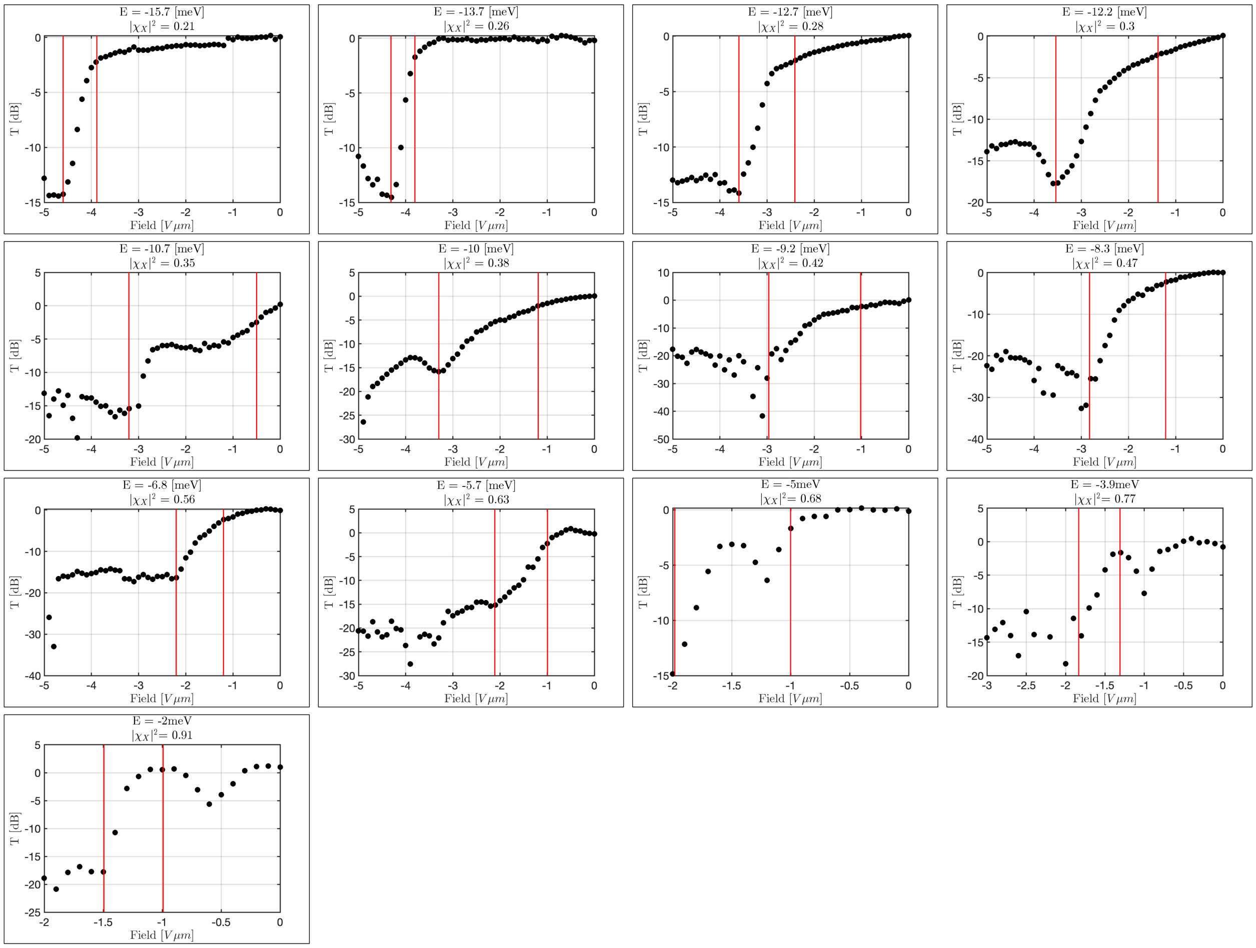

Figure S3: Raw Data of the switching performed with DC electric field, used in Fig. 1(g and h)

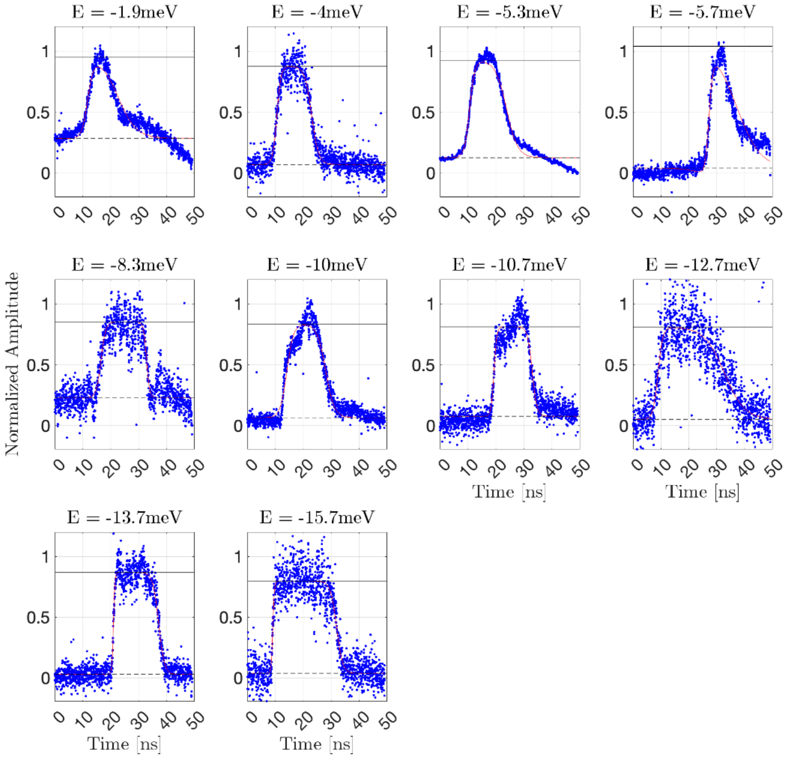

Figure S4: Raw Data of the switching performed with a pulsed electric field, used in Fig. 2(e-f)

References

(1) Liran, D.; Rapaport, R.; Hu, J.; Lydick, N.; Deng, H.; Pfeiffer, L. Electrically Controlled Photonic Circuits of Field-Induced Dipolaritons with Huge Nonlinearities. *Physical Review X* **2024**, *14*, 031022.

(2) Rosenberg, I.; Mazuz-Harpaz, Y.; Rapaport, R.; West, K.; Pfeiffer, L. Electrically controlled mutual interactions of flying waveguide dipolaritons. *Physical Review B* **2016**, *93*, 195151.
